# Nanosilicon-Based Composites for (Bio)sensing Applications: Current Status, Advantages, and Perspectives

**DOI:** 10.3390/ma12182880

**Published:** 2019-09-06

**Authors:** Valerii Myndrul, Igor Iatsunskyi

**Affiliations:** NanoBioMedical Centre, Adam Mickiewicz University, 3, Wszechnicy Piastowskiej Str., 61-614 Poznan, Poland

**Keywords:** silicon, nanomaterials, (bio)sensors, nanocomposites

## Abstract

This review highlights the application of different types of nanosilicon (nano-Si) materials and nano-Si-based composites for (bio)sensing applications. Different detection approaches and (bio)functionalization protocols were found for certain types of transducers suitable for the detection of biological compounds and gas molecules. The importance of the immobilization process that is responsible for biosensor performance (biomolecule adsorption, surface properties, surface functionalization, etc.) along with the interaction mechanism between biomolecules and nano-Si are disclosed. Current trends in the fabrication of nano-Si-based composites, basic gas detection mechanisms, and the advantages of nano-Si/metal nanoparticles for surface enhanced Raman spectroscopy (SERS)-based detection are proposed.

## 1. Introduction

Nanoscale (porous) silicon (Si) was accidentally discovered in 1956 by Arthur Uhlir Jr. and Ingeborg Uhlir in the process of developing a technique for polishing and shaping the surface of silicon [1]. However, for a long time, this material was beyond the concerns of the scientific community until A. G. Cullis and L. T. Canham reported on the visible light emission due to the quantum size effects in highly porous crystalline silicon (PSi) in 1990 [2]. This discovery provided another opportunity for further investigation and application.

Up until now, nano-Si remains one of the most popular and sought-after materials in applied science. The fabrication procedure of nanoscale silicon is not labor intensive and does not require special (expensive) equipment and chemicals. Depending on the structure/morphology, for example, porous silicon (PSi) [3,4,5], silicon nanopillars (SiNPs) [6,7], and silicon nanowires (SiNWs) [8], this material can be used for Li-ion batteries [9], water-splitting [10], solar cell [11], sensor and biosensor applications [12,13], etc.

(Bio)sensors are devices designed for the selective detection of (bio)molecules in a multimolecular environment. Generally, they consist of a detection platform (transducer) with a selective layer and target (bio)molecules in liquids or gases. The main idea is to observe the modification of the transducer response (optical, electrical, chemical, thermal, etc.) through “surface–target analyte” interaction in real-time or express detection [14].

Nowadays, sensors and biosensors based on nano-Si have been successfully applied to molecules [15], biomolecules [16] and light [17] detection using different responses (PL [18,19], SERS [20], I–V [21], reflectance [22,23], resistance [24], capacitance [25], fluorescence [26]) and material modifications (PSi, SiNWs, SiNPs). Such strong interest in (bio)sensors based on nano-Si can be explained by their enhanced surface to volume ratio, biocompatibility, and low-cost.

The most common methods for PSi sample fabrication are metal-assisted chemical etching (MACE), stain etching, and electrochemical etching [27]. Use of these methods enables the fabrication of PSi substrates with different pore sizes (from nanoporous to macroporous), depending on the chemical/physical procedure parameters. Currently, many works have been dedicated to PSi-based (bio)sensor application as well as PSi-based nanocomposites (PSi/Au [13], PSi/ZnO [8], PSi/TiO_2_ [28,29,30]) with enhanced selectivity, sensitivity, and tailored properties. 

SiNWs and SiNPs are the most advanced modifications of nano-Si due to their enhanced surface to volume ratio when compared with PSi. On the other hand, their fabrication involves additional steps such as etching mask deposition by using photolithography [31], polystyrene nanosphere lithography [6], or electron-beam lithography [32]. Recently, attention from the scientific community has been given to the fabrication of highly-sensitive (bio)sensor platforms based on SiNW and SiNP nanocomposites. It has been established that Au, Ag, Pd, and Pt nanoparticles deposited over silicon nanopillars or nanowires can be aggregated to “hot spots” and demonstrate a high enhancement factor in SERS-based biosensors with a detection limit less than 10^-12^ M [33]. Furthermore, SiNWs and SiNPs in conjunction with metal oxides (TiO_2_, ZnO, WO_3_, F_2_O_3_, TeO_2_) have shown promising results for gas and biomolecule detection via an electrochemical response with a detection limit of about 1 ppm [34,35,36,37,38]. Recently, a number of new composites have been developed based on SiNWs and SiNPs with sulfides (CdS, MoS_2_) [39,40] and nitrides (Si_3_N_4_) [41] that are suitable for sensitive light, humidity, and gas detection due to enhanced absorption and adsorption.

Tailored and advanced properties of nano-Si and silicon nanocomposites open great possibilities for use in novel trends in (bio)sensor applications. This paper is dedicated to nano-Si and silicon nanocomposites suitable for (bio)molecule detection as well as future prospects of this research area. Additionally, the application of nano-Si and its nanocomposites for (bio)sensors was discussed. The effects of metal and metal oxide nanoparticles on the structural, optical, electrical, and (bio)sensor properties were analyzed. The mechanism of interaction between nano-Si/silicon nanocomposites and (bio)molecules was also clarified. New trends, affecting the development of nano-Si-based biosensors are presented.

## 2. Types of Nano-Si Morphology and Methods of Fabrication

### 2.1. Porous Silicon (PSi)

**Figure 1:**
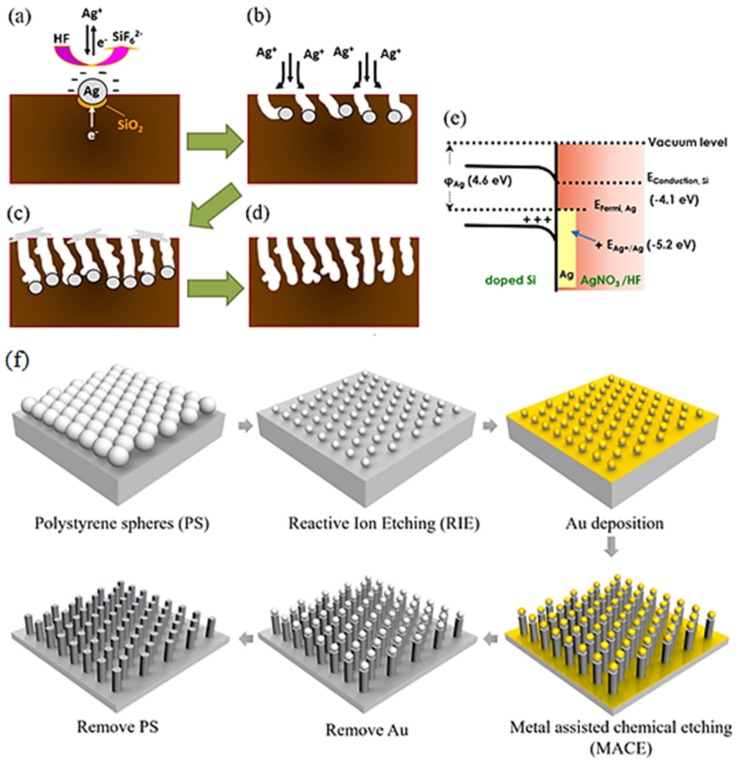
(**a**–**d**) Schematic illustrations of the formation mechanism for synthesizing porous Si films using the MACE process [50]. (**e**) Electrochemical energy diagram of corresponding reaction. The illustration of the Si NPAs fabrication process [50]. (**f**) Schematic illustration of the fabrication of SiNP arrays. Close-packed monolayer of polystyrene (PS) nanospheres on a clean Si reduced diameter of PS by reactive ion etching, Au deposition, metal-assisted chemical etching, and the removal of Au/PSi [51].

**Figure 2:**
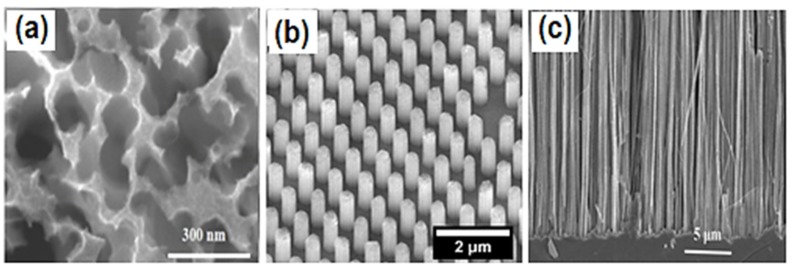
Scanning electron microscopy images of PSi (**a**) [4], SiNPs (**b**) [10], and SiNWs (**c**) [52].

PSi is a well-studied Si-based nanomaterial. As above-mentioned, PSi has obtained great interest within the scientific community after light emission was discovered in 1990. PSi has a number of unique properties such as visible light emission, enhanced light absorption, and biocompatibility. Recently, a number of publications have been dedicated to PSi and PSi-based nanocomposite fabrication and its application in (bio)sensing. As previously mentioned, electrochemical anodization, stain etching, and MACE (Figure 1a–d) [42] remain the most common methods for PSi substrate fabrication, which enable the production of PSi (Figure 2a) with tailored morphological properties (porosity, pore size, and depth of pores).

### 2.2. Silicon Nanowires (SiNWs)

SiNWs (Figure 2c) are another type of nano-Si, where the height of the Si nanoelements is much higher than its diameter (h >> d). Due to the high surface to volume ratio, SiNWs have found successful applications in solar cells, sensors, biosensors technologies, photovoltaics, etc. [43]. Traditionally, this nanomaterial can be fabricated from bulk Si by RIE [44] and MACE [45] in combination with lithographic techniques (photolithography, polystyrene nanosphere lithography) or bottom-up and top-down technologies [46]. In addition, the initial synthesis of SiNWs is often accompanied by thermal oxidation steps to yield structures with an accurately tailored size and morphology [47].

### 2.3. Silicon Nanopillars (SiNPs)

A SiNP (Figure 2b) substrate (h ≥ d) is a kind of nano-Si with densely packed and well-ordered morphology. This substrate, like that of SiNWs, possesses an enhanced surface to volume ratio and absorption when compared with bulk silicon. Relying on this fact, SiNP arrays have become popular and prospective for solar, cell water-splitting, and (bio)sensors application. This kind of nano-Si is generally fabricated by RIE and MACE with different types of lithographic masks (Figure 1f) [48,49]. The mechanical robustness of the SiNP area is substantially better when compared with SiNWs due to h~d and a well-ordered morphology.

## 3. (Bio)sensors Based on PSi, SiNWs, SiNPs and Their Composites with Polymers

Nowadays, nano-Si remains one of the most popular materials for sensor and biosensor applications. A number of unique properties make it prospective for (bio)molecules, pH, and light detection via different sensing techniques (optical, resistive, volt-amperometry, etc.). High surface to volume ratio allows for an increase in the number of adsorbed (bio)molecules, resulting in enhanced sensitivity when compared with planar Si surfaces. The selectivity of nano-Si to the target analyte can be achieved via (bio)functionalization such as a bioselective layer for target biomolecules (e.g., antigen–antibody interaction) [3,19]. Additionally, significant interest by the scientific community has been paid to real-time measurements and the design of a microfluidic system with embedded nano-Si transducers [53].

As mentioned below, biofunctionalization plays a very important role in bioselective layer evolution and allows for the binding of organic molecules to a non-organic nano-Si surface without unspecific interaction. Currently, a number of biofunctionalization protocols have been proposed: silanization [3,19,53,54,55,56,57,58,59,60,61,62,63,64,65,66,67], aminosilanization [68,69,70], direct immobilization [16,22,71,72], enzyme [18] or peptide [73] treatment, phospholipid bilayers formation [74], hydrosilylation treated by N-Hydroxysuccinimide and 1-Ethyl-3-(3-dimethylaminopropyl)carbodiimide (NHS/EDC) [75,76,77] or resazurin [78], and polymer synthesis [79]. However, the most common technique is silanization, due to the possibility of controlling the thickness of the(3-Aminopropyl)triethoxysilane (APTES) layer as well as using different cross-linking agents (glutaraldehyde, NHS/EDS) [18,80].

In recent years, nano-Si has been widely used for optical (bio)sensor applications due to its portability and high sensitivity. Among all of the optical detection approaches, photoluminescence (PL)-based measurement looks the most promising, especially for real-time monitoring [3,18,19,72,78,81,82,83,84]. Previously, we reported on low-cost, highly sensitive PSi-based immunosensors for ochratoxin A (OTA) detection using a PL approach. It was established that the intensity of PL changes under different OTA concentrations via antibody–antigen interaction onto the PSi surface. The limit of detection (4.4 pg/mL) and the sensitivity range (0.01–5 ng/mL) to OTA were estimated [3,19]. In [18], Syshchyk et al. reported on a PSi-based photoluminescence platform for heavy metals, urea, and glucose detection. PSi surface biofunctionalization was performed by enzyme (urease and glucose oxidase) treatment. The sensor mechanism was based on the effect of PL changing with the varying pH of the solution caused by the enzymatic reactions [18]. Furthermore, it was reported that the PL-based detection approach could be utilized for O_2_ detection on a SiNW platform [84]. SiNWs were fabricated by the MACE method and O_2_ detection was carried out through the measurement of different oxygen flow pressure. The general sensing mechanism was based on the PL intensity change, which can be explained by the reversible charging/recharging of surface defects (Pb-centers) due to the oxygen adsorption/desorption.

Another nano-Si optical response suitable for (bio)molecule detection is reflectance or other optical parameters related to reflectance [22,48,53,55,56,57,58,62,65,67,68,70,71,74,76,85,86,87,88,89,90,91,92,93,94,95,96]. Generally, the (bio)sensor technique based on reflectance response can be performed via reflective index (RI) [71] or optical density [16] (OD) measurements in the initial state and after the addition of the analyte. The changes in RI and OD caused by analyte-transducer surface interaction can be processed and used as the effective (bio)sensor signal. Other pathways for detection based on reflectance usually involve the analysis of the interferogram average over wavelength (IAW–IAW_0_) [89,97] as well as the estimation of effective optical thickness ratio (EOT/EOT_0_) [53,54]. For instance, PSi sensors based on the reflectance response for heavy metal detection were studied in [61,97,98,99]. Politi et al. reported on the highly-sensitive (LOD ~ 1.2 ± 0.3 ppb) method for Pb(II), As(III), and Cd(II) detection via the modification of PSi surfaces by lysine and oligopeptides [98]. The advanced optical approach for *E. coli* detection was also proposed by Y. Tang et al. [53]. Real-time measurements were performed in a microfluidic system with a PSi oxidized substrate via indirect Fourier transformed reflectometric interference spectroscopy (FT-RIS) measurements. Detection included two steps: capture of the bacteria on the PSi surface and measurement of pore accessibility by BSA treatment. It was assumed that the EOT shift of PSi decreased with increased *E. coli* concentration on its surface, causing a block of the porous array. Furthermore, Luan et al. developed photonic waveguides and microring resonators based on SiNPs for a high sensitivity label-free transducer that was suitable for isopropyl and streptavidin detection [71]. The sensitivity of each resonator to isopropyl (228–580 nm/RIU) was calculated as the ratio of the wavelength shift slopes to the change of reflective index (RI). The authors noted that sensitivity could be enhanced by minimizing the scattering loss by applying the new advanced fracturing strategies and single line edge smoothing (SLS) in the process of nano-Si fabrication.

**Figure 3:**
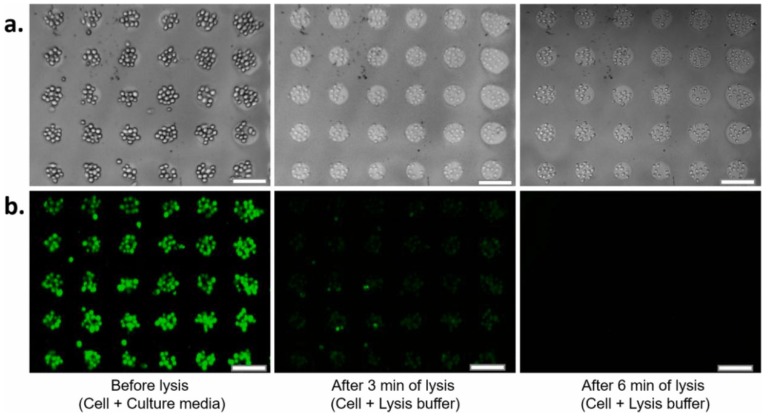
(**a**) Bright field (BF) and (**b**) fluorescence images of J774 macrophage cells on pattern before and after lysis. The dye for cells staining was calcein AM. When the cells were lysed, pores were created on the cell membrane, thus causing the leakage of calcein from the cells. Thus, the fluorescence intensity started to decrease due to the leakage of calcein. Cells were still on the micropatterns after lysis, as can be seen from the BF images. Scale bar 100 μm [75].

Fluorescent optical response is usually used for the labeled biomolecule detection technique [60,64,66]. The general idea of this approach is based on analysis of a fluorescence signal from labeled biomolecules via their binding with previously functionalized nano-Si structures. In [64,66], the PSi Bragg mirror was used to enhance the fluorescence signal from the CdSe/ZnS QD embedded within the PSi pores for single-stranded DNA (ssDNA) detection. Target DNA hybridization was labeled with a cyanine (Cy3) fluorophore and the detection limit to DNA hybridization was estimated as 1 nM [60]. The novel “label-free” fluorescent detection approach was proposed by Piya and coauthors [75]. Arginylglycylaspartic acid (RGD) peptides have been used to provide non-selective adhesion of target J774 macrophage cells on (polyethylene glycol) PEG hydrogel patterned PSi Bragg reflectors. The J774 cells previously stained by calcein AM and adhered over peptides were lysed chemically. When the cells were lysed, there was a leakage of calcein from inside the cells due to the rupture of the cell membrane that led to a decrease in fluorescence intensity (Figure 3). This approach was suitable even for single cell detection, however, the selective layer was not described [75].

**Figure 4:**
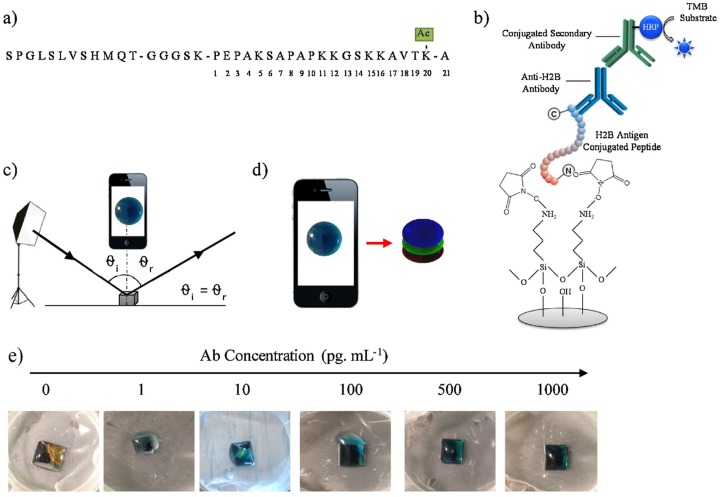
(**a**) The sequence of the 21-mer Si-specific peptide conjugated with the H2 B antigen (the site of acetylation is annotated); (**b**) Schematic representation of the H2 B glass sensor; (**c**) The measuring scheme, (**d**) the red-green-blue (RGB) layers of the obtained colored product; (**e**) Generation of colored solution by TMB-HRP reaction after capture of H2 B antibody on PSi. Color intensity depends on the concentration of the captured Anti-H_2_ B antibody [73].

In [73,100], the authors reported on the visual colorimetric sensing techniques suitable for (bio)molecule detection. Photonic polymer modified PSi templates have shown prospective results for non-pathogenic *E. coli* and isopropanol alcohol detection. The key idea for the development of composite sensors capitalized on the high refractive index contrast afforded by Si. It was established that composite sensors gave a strong reflectance spectrum that was more readily seen by the eye when the sensor was wetted with the isopropanol solution. These photonic PSi/polymer composites have also shown enhanced sensitivity to *E. coli* when compared with all-polymer photonic sensors. This can be attributed to differences in their wettability, which affects *E. coli* adhesion [100]. Ramakrishan et al. reported on a PSi microcavity for autoimmune disease detection based on H_2_ B antigens or antibodies quantification via red, green, and blue (RGB) spectral analysis (Figure 4). Images for RGB analysis were captured by smartphone camera and blue color information was extracted. An extremely low concentration (10 fg/mL) of autoimmune antibody was detected, making this approach suitable for application [73].

Optical transmittance of PSi microring resonators and microcavities was used as the signal for sensor and biosensor applications [101,102,103,104]. Weiss et al. reported on 10 μm and 25 μm microring waveguides for nucleic acid (PNA) detection via transmittance measurements. It was established that PNA hybridization shifts the resonance peak at 2.00 nm and 1.48 nm for the 10 μm and 25 μm radius PSi rings, respectively. This difference in resonance shift with PNA treatment can be explained by the variation in molecular adsorption on the two samples [101,102]. Girault et al. proposed a similar approach for glucose quantification in aqueous solutions. Despite the fact that the LOD was estimated as 0.7 g/L, information about the selectivity to glucose was not available [103].

In parallel with the above-mentioned optical transducers, nano-Si is widely used for (bio)sensor application based on electrical and electrochemical responses [77]. For instance, I(J)-V measurements were carried out for the detection of biomolecules [79,105], gases [21,49,106,107,108], light [109,110,111], and pH [112,113,114]. Shashaani et al. reported about Mebendazole (MBZ) drug activity on breast cancer cells (MCF-7) adhered over a SiNW chip [105]. It was established that MCF-7 cells treated with MBZ drugs caused a significant (increased from 5 nA to 300 nA for 2 nM of MBZ) effect on I–V patterns due to the change in the ionic state of cytoplasm, and subsequently, the ionic equilibrium between the cell’s inner and outer parts. The detection limit to the MBZ drug tracing was calculated as 0.01 nM [105].

Capacitive [21,115,116] and resistive [45,115,117,118] responses of the nano-Si substrates were examined for gas and alcohol detection. Qin et al. reported on enhanced H_2_ adsorption on SiNWs fabricated by MACE and post-etched in KOH to enhance the surface rough. It was shown that relative resistance response to 200 ppm H_2_ was equal to 83% and significantly higher than for the same concentration of methanol, ethanol, isopropanol, acetone, or methane at room temperature [45]. In addition, Qin et al. reported on Polypyrrole (PPy) shell/Np functionalized SiNWs (PPy-shell@SiNWs and PPy-NPs@SiNWs) suitable for ultra-low detection resolution (130 ppb) and excellent selectivity toward NH_3_ [118]. The underlying mechanism for the enhanced relative resistance response of PPy-shell@SiNWs in comparison to the PPy-NPs@SiNWs was analyzed based on the modulation of PPy sensitization on axial conductance. In [115], PSi sensing elements on paper for humidity sensing were demonstrated. The detection approach was based on the relative resistance and capacitance measurements in environments with different humidity. The PSi based humidity sensor was used for real-time measurements and a relatively fast recovery was observed even though no refreshing methods were employed.

Thual et al. proposed a theoretical model of hybrid Psi–polymer optical waveguides for BSA detection [119]. Due to the PSi high specific surface and biocompatibility, it was used as the sensing part of the sensor. Additionally, polymer waveguides were fabricated for the reference part of the sensor due to their low optical losses. The theoretical limit of detection and sensitivity were calculated as 0.019 pg mm^−2^ and 12.5 nm/(pg mm^−2^), respectively.

## 4. (Bio)sensors Based on Nano-Si and Metals Oxides Nanocomposites

Currently, there is a growing number of publications dedicated to the (bio)sensing properties of nanocomposites based on nano-Si and metal oxide (MOx). Such significant interest in these types of nanomaterials can be explained by the enhanced sensitivity [17,24,120,121] and surface stability [25,26,122] of these nanocomposites. MOx nanoparticles and nanolayers synthesized over nano-Si can positively effect nano-Si surface passivation and degradation. The advances in nano-Si fabrication and MOx deposition enable the production of nanocomposites with tailored morphologies and electro-optical properties (photoluminescence, type of conductivity, etc.), which play a crucial role for the effective detection of (bio)molecules. Mainly, MOx nanolayers/nanoparticles can be deposited over a nano-Si surface through the following techniques: (i) RF and DC magnetron sputtering [24,34,36,37,120,121,123,124,125,126]; (ii) sol–gel/hydrothermal synthesis + spin coating [17,26,127,128,129,130,131]; (iii) drop casting technique + pulsed laser ablation in liquid [132]; (iv) vapor–liquid–solid growth and chemical vapor deposition [25,40,133]; (v) catalytic immersion method [134]; and (vi) electrochemical and chemical deposition [35,122,135].

**Figure 5:**
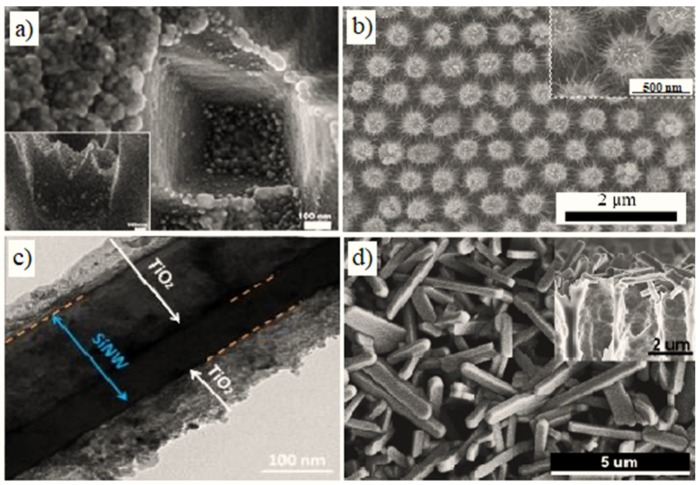
Scanning electron microscopy (SEM) images of some nano-Si/MOx nanocomposites: (**a**) SEM images of the PSi/ZnO nanocomposite [125]; (**b**) SEM images of the SiNWs/WO_3_ nanocomposite [126]; (**c**) SEM image of the SiNPs/TiO_2_ nanocomposite [136]; (**d**) SEM images of the PSi/V_2_O_5_ nanocomposite [37].

Some types of nano-Si/MOx nanocomposites used as a (bio)sensor platform are shown in Figure 5.

It has been ascertained that silicon/MOx nanocomposites are widely used for gas detection through the I–V curve characterization [136], resistance [24,34,35,37,39,120,121,122,124,125,126,129,131,133,135,137,138], and capacitance [25,40] measurements. Generally, the main gas sensing mechanism is based on oxygen adsorption on the nano-Si/ MOx surface, causing electron extraction from the conductive band of semiconductors. This leads to a reduction in the electron concentration and hence the initial resistance increase or decrease for p-type and n-type semiconductors, respectively [37]. In the next step, chemisorbed oxygen species react with different molecules (H_2_, CO_2_, ethanol, acetone, isopropanol, toluene gas, etc.), releasing the electron back to the conductive band of the semiconductor, and causing a reverse change in resistance.

It was found that p-p and p-n heterojunctions formed at the interface of nano-Si/MOx nanocomposites play an important role in charge separation and charge life-time increasing due to the barrier layer formation. Liu et al. proposed that the composition of p-CuO and p-PSi led to a p-p heterojunction formation due to the different electron affinity (χ(CuO) = 4.07 eV, χ(PSi) = 4.01 eV) [124]. As the Fermi levels are not at the same level, electrons from CuO migrate to Psi, and holes migrate in the opposite direction until the Fermi energies become equal. This charge transfer leads to a formation of the depletion layers in PSi and CuO, respectively. The heterojunction effectively separates charges, resulting in the high concentration of holes in the accumulation layer and increased the lifetime of the charge carriers. This simplifies the electrons extracted from the conductive band of heterostructures during the gas adsorption. A similar mechanism was proposed for p-TiO_2_/p-PSi [34], p-Cu_2_O/p-PSi [135] and proven by experimental measurements.

**Figure 6:**
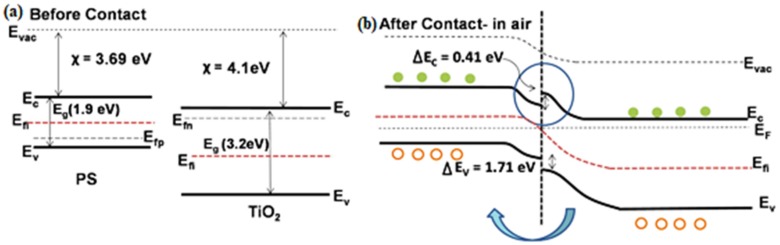
Band diagram of the TiO_2_ decorated PSi heterojunction (**a**) before contact, (**b**) after contact (in air) [120].

A number of works have also been published on the p-n heterojunction by using a combination of p-type PSi and n-type ZnO [24,35,36,122,125,134], WO_3_ [36,129,137,138,139], SnO_2_ [122,133], V_2_O_5_ [37], and TiO_2_ [120]. The sensitivity of these nanocomposites was enhanced in comparison to the bare semiconductors and this can be explained as follows [120]: (a) a reduction in the surface activation energy E_a_ upon the formation of the p-n heterojunction, resulting in increased analyte adsorption; (b) the presence of oxygen species and dangling bonds on PSi/MOx, and as a consequence, more reaction sites on the surface, which improved the adsorption of target molecules. As an example, Figure 6 shows the band diagram of TiO_2_/PSi. The formation of the heterojunction produces the barrier effect, so electrons lose their capacity to move from the n to p side. In this case, the holes play a main role in sensing. When the surface of the nanocomposites is exposed to air, the number of holes on the surface increases (Equation (1)) [120].

1/2 O_2_ (g) → O^−^(ads) + h^+^, (1)

when the sensor is treated with some gases, free electrons are injected to the surface, and neutralized holes result in an increase in sensor resistance.

It should be noted that tuning the scale of the MOx nanolayer or nanoparticles and the morphology of the Si surface are very important elements for sensor design. Husairi et al. showed that the PSi/ZnO sensor response to ethanol depends on the concentration and type of defects and area of active sites for absorption as the number of defects and active species on the PSi/ZnO surface was directly affected by the precursor (Zn(NO_3_)_2_6H_2_O) concentration [134]. In [122,125], ZnO nanolayers were deposited over PSi and c-Si by using zinc acetate (ZA) and carbonate (ZC) precursors via chemical bath deposition (CBD) and the magnetron-sputtering technique, respectively. It was demonstrated that PSi/ZnO possessed enhanced sensitivity in comparison to c-Si/ZnO. This was due to the increase in the PSi/ZnO effective surface area, resulting in higher adsorption on its surface [125]. On the other hand, the PSi/ZnO substrate deposited using ZC showed a better response to CO_2_ than film deposited using ZA due to a more homogeneous covering [122].

Nano-Si/MOx nanocomposites have been applied as biosensors [26,130,140]. In [26], PSi/TiO_2_ substrates showed enhanced sensitivity to mycotoxins in comparison with pure PSi. Before the sensing experiment, PSi/TiO_2_ and Psi were functionalized by (3-Glycidyloxypropyl)trimethoxysilane (GPTMS) and selectivity to the mycotoxins was achieved by using hybridized aptamers of mycotoxins. Furthermore, both substrates were exposed to the same concentration of Cy3-labeled mycotoxins and fluorescence intensities were collected by utilizing a fluorescence scanner. It was found that the fluorescence intensity of the analyte on the PSi/TiO_2_ surface was almost 14 times higher than the thermally oxidized PSi surface. This result can be attributed to the following reasons: (i) the surface of PSi/TiO_2_ was more stable than PSiO_2_; and (ii) the surface of PSi/TiO_2_ had more active sites for analyte immobilization. The emission intensity of the dye was increased because the polar TiO_2_ surface enhanced the delocalization of the *π* electrons and lowered the highest occupied molecular orbital and lowest unoccupied molecular orbital energy levels of the dye [26].

**Figure 7:**
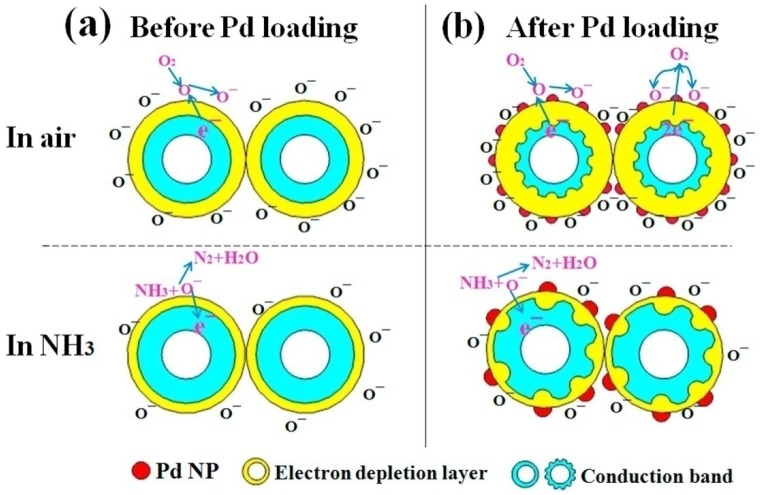
Mechanism diagram of PSi/WO_3_/Pd to NH_3_: (**a**) Before Pd loading, (**b**) After Pd loading [139].

The sensitivity of nano-Si/MOx via noble metal deposition [15,36,38,121,139,141,142,143] has also been studied. It a found that noble metal (Ag, Au, Pt, Pd) nanoparticles, imbedded into nano-Si /MOx nanocomposite play an important role in charge generation and significantly increases the quantity of the chemisorption of oxygen ions O^−^ and creates additional active sites, leading to the formation of a deeper depletion region in comparison to that of pure sensors [80,112,115]. Herein, Qiang et al. reported on enhanced sensitivity of PSi/WO_3_/Pd nanocomposites to NH_3_ [139] (Figure 7a) and NO_2_ [15] gases. The main differences between the PSi/WO_3_/Pd and PSi/WO_3_ sensing mechanisms were explained by the following (Figure 7b,c) [139]:

- In the case of the PSi/WO_3_ nanocomposite, the sensing mechanism directly depends on the heterojunction parameters and efficiency of O_2_ absorption-desorption;
- PSi/WO_3_ substrates decorated with Pd NPs would possess enhanced catalytic activity that will lead to enhanced dissociation of oxygen molecules O_2_ and absorption of oxygen ions O^−^ on the PSi/WO_3_/Pd surface. More ion absorbed oxygen on the surface would provide more sensing sites, leading to enhanced gas response and reaction rate.
- Additionally, the work function of Pd was larger than that of WO_3_, therefore the electrons from WO_3_ will transfer to Pd, causing the generation of the Schottky barrier at the interface between Pd and WO_3_. By these reasons, the conduction band of PSi/WO_3_/Pd will become much narrower when compared with WO_3_ and the concentration of the conduction electrons will be reduced. As a consequence, the interaction of NH_3_ molecules with the PSi/WO_3_/Pd substrate will lead to more significant resistance variation and higher sensor response.

## 5. (Bio)sensors Based on Nano-silicon and Metals Nanoparticles

The large active surface of nano-Si as well as enhanced stability, catalytic activity, and surface-enhanced Raman scattering (SERS) of the metal nanoparticles in combination are very promising for highly-sensitive (bio)sensor applications. Therefore, different nano-Si/metal nanocomposites (MNps) have been widely employed for rationally designing and fabricating high-performance (bio)sensors for the detection of various chemical and biological species [144]. The deposition of metal nanoparticles/nanofilms over all types of nano-Si can be implemented by the following techniques: (i) magnetron sputtering [31,51,145,146,147,148,149]; (ii) immersion, chemical, and electrochemical depositions [13,20,27,150,151,152,153,154,155,156,157,158,159,160,161,162,163,164,165,166,167,168,169,170]; (iii) thermal evaporation [32,44,171,172,173,174,175,176,177,178,179]; and (vi) laser ablation technique/pulsed laser deposition [180,181].

Nowadays, nano-Si/MNps nanocomposites have been utilized for (bio)sensors based on SERS [12,20,31,32,51,145,149,150,151,152,153,154,155,165,168,173,175,176,177,178,182,183,184], optical [13,44,158,164,167,171,180], and electrical [27,146,148,156,159,160,161,162,166,169,170,172,173,179,181,185,186] responses. Among all of these approaches, SERS of MNps decorated nano-Si is extensively exploited as the most efficient spectroscopic phenomenon for high-sensitive sensing. The development of a practically applicable SERS-based (bio)sensor requires an efficient SERS substrate, which possesses strong enhancement factors (EF), robustness, stability, uniformity, and reproducibility. It was found that PSi has a major flaw for these applications because the surface morphology has an uncontrolled stochastic character, making it impossible for hot spots to be uniformly distributed over the surface [51,177]. Therefore, 3D nano-Si substrates such as SiNPLs and SiNWs are more suitable for SERS-based (bio)sensors because of their well-ordered surface, leading to uniform distribution and the accessibility of hot spots (see Section 2). Furthermore, arrays of SiNPLs and SiNWs stabilize the distribution of MNps, which results in high EF and excellent reproducibility with a low detection limit [149]. For instance, in [31,51,149,177], 3D SiNPs/Ag and SiNPs/Au nanocomposites were utilized for Rhodamine 6G (R6G) molecule detection via SERS. The authors showed that the smallest limit of R6G detection was equal to 10^−13^ M [149]. This was attributed to the high EF (2.4 × 10^8^) achieved due to the well-organized fabrication and variation of wavelength excitation.

In order to obtain a high-sensitive SERS–active platform, the authors in [20] proposed a multi-step fabrication process including the following steps: (i) fabrication of Ag dendrites; (ii) AuNPs deposition over Ag dendrites; (iii) synthesis of Si nanoneedles; and (iv) nanoneedle decoration by AgNPs. The authors noted that such 3D multi-structures were fabricated to achieve a much stronger enhancement when compared with the SERS-active AgNPs or 1DAg dendrites. Additionally, the hierarchical scaffolds and the hydrophilic performance could endow the substrates with improved sensitivity and reproducibility. Eventually, the substrates showed a low limit of detection to malachite green (~10^−13^ M), which may be promising in the field of sensing, imaging, and clinical diagnosis.

**Figure 8:**
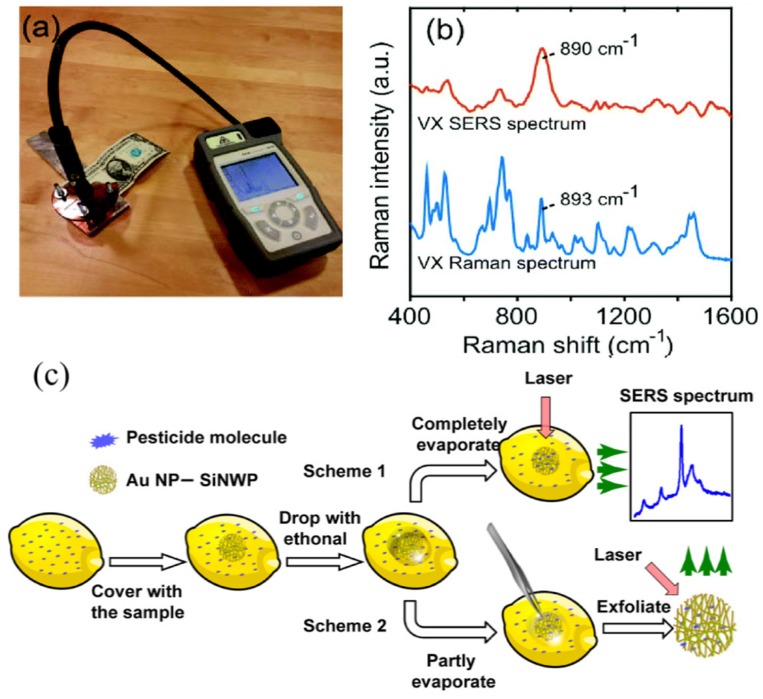
(**a**) The handheld Raman instrument. (**b**) SERS spectrum of 0.75 nmol VX and normal Raman spectrum of >98% VX solution [12]; (**c**) pathways for the in situ detection of pesticide residues on lemon peels using flexible SiNPs/Au [184].

In [12,184], SERS measurements were applied for real sample investigation. Hakonen et al. constructed a handheld (Figure 8a,b) device based on the SiNWs/Au SERS signal for polar organic liquids O-ethyl S-(2-diisopropylaminoethyl) methylphosphonothiolate (VX) and Tabun detection at ambient conditions [12]. The low detection limits were achieved for nerve gases due to high droplet adhesion. The high sensitivity result of the small droplet contact area and target molecule accumulation within the SERS hot-spots were formed by clustered nanopillars. Cui et al. reported on flexible, transparent, and self-standing SiNWs/Au consisting of ultrathin three- dimensional SiNW networks suitable for pesticide residue detection via SiNWs/Au wrapping onto the lemon surface [184]. SERS signals were collected by two approaches: (i) directly, from the lemon surface with a previously adhered small piece of SiNWs/Au and treated with ethanol; (ii) SiNWs/Au paper could be torn off the lemon surface before the ethanol completely evaporated and the Raman signal could be recorded from the sample placed on a flat Si substrate or glass. The limit of detection to pesticides on the lemon surface was estimated as 72 ng/cm^2^ for both approaches, meaning that this technique has the potential for fast in situ and nondestructive sensing (Figure 8c).

In [52,181], SiNWs/Pt/Pd and SiNWs/Pd were used for H_2_ detection via resistance and I–V measurements, respectively. It was suggested that H_2_ physical and chemical adsorption on Pt/Pd nanoparticles takes place through the incorporation of hydrogen atoms into a metal lattice (MH_x_) [181]. Physisorbed molecules on the nanoparticle’s surface and H species incorporated in the interstitial sites of the Pt/Pd NPs can act as electron scattering centers and decrease the carrier mobility, causing an increase in the electrical resistance of the Pt/Pd ultra-thin film. When Pt/Pd is deposited over the SiNWs, it is also will take the place of the shortest current path by contacting the neighboring clusters and thus perfect contacts can be formed between almost all nanowires inside each cluster at higher H_2_ concentration ranges. For this reason, after hydrogen absorption, electron scattering was reduced and the resistance change was rapid, this phenomenon forms the basis of H_2_ detection. Such a point of view has correlation with the results published in [52]. In the process of the H_2_ deposition over SiNWs/Pd, they dissociated into hydrogen atoms, causing the I–V curve to shift and a significant reduction in the current. These processes can be explained by the SiNWs/Pd Schottky barrier increasing (from 0.678 meV to 0.685 meV) when H_2_ was adsorbed. It was noted, that according to the Butler theory, the absorption and desorption of H_2_ in a thin layer of Pd at room temperature and pressure leads to the reversible hydride PdH_x_, where x is the atomic ratio H/Pd [52]. The absorption of H_2_ can be related to a crystallographic phase transition.

In our previous research [13], we showed that Au nanoparticles deposited onto the PSi surface led to an increase in the sensitivity to the target (Aflatoxin B1) and decreased the response time of the immunosensors. The analytical performance of the PSi/Au PL-based immunosensor showed very good characteristics with a maximal sensitivity range within 0.01–10 ng/mL. Compared to the standard enzyme-linked immunosorbent assay (ELISA) [3] method, the Au/PSi immunosensor showed about 100 times lower concentration range. In [180], PL-based sensing was performed for ethanol, n-hexane, and trichloroethylene detection on a PSi/Au platform. It was found that the PL intensity of the PSi/Au nanocomposite in ethanol vapor was significantly less compared with the PL intensity in n-hexane and/or trichloroethylene. This can be attributed to the larger dipole moment in ethanol, leading to the enhancing of non-radiative emissions in the PSi/Au surface layer.

Cui et al. reported on the 2D PSi/Au platform for explosives detection and identification [164]. The main idea of this approach was based on the simultaneous measurements of PSi/Au electroluminescence (ELC) peak intensity and position under interaction with explosives including nitro compounds, peroxides with nitrogen atoms, and peroxides without nitrogen atoms due to their different oxidation and electron transfer ability. In this case, Au nanoparticles catalyze the oxidation reaction between PSi and H_2_O_2_ and due to this, the ELC change is faster in comparison with bare PSi. Consequently, it was established that pre-oxidation of PSi with oxidants could introduce surface defects and, accordingly not only quench the ECL intensity, but also decrease the rate of the initial peak shift when compared with the blank PSi. In contrast, explosives containing the nitro group could just quench the ECL of PSi through the electron transfer process but without a pre-oxidative effect, whereas compounds with an electron donating ability (e.g., amine group) could enhance the ECL intensity. However, if this compound also contains a peroxy group, the quenching and enhancing effect might be counteracted.

## 6. (Bio)sensors Based on Nano-Si and Carbon-based Nanomaterials

As previously mentioned, the current trends in (bio)sensors are oriented toward the development of novel composite nanomaterials in order to obtain sensing substrates with enhanced surface to volume ratio, biocompatibility, and sensitivity. In the last decade, carbon based materials (carbon nanotubes (CNT), graphene (G), graphene oxide (GO)) have recommended themselves as efficient platforms suitable for (bio)sensor applications due to their high electron mobility, large surface area, and biocompatibility. Therefore, it is expected that materials based on carbon nanomaterials incorporated with nano-Si will possess more efficient sensing with a wide detection range and low detection limit. Another advantage lies in the fabrication process, which is not labor intensive and not time consuming, for instance, graphene can be synthesized over nano-Si through the in situ CVD process [187]. In [188,189], fabrication processes were carried out by the separate preparation of nano-Si and graphene substrates with the following graphene transfer on the nano-Si surfaces. In the case of graphene oxide, it can be covalently bonded to the PSi in the presence of EDC/NHS [190] and added dropwise over the substrate followed by spin coating [191].

Currently, nano-Si/carbon-based nanomaterials have been examined as (bio)sensor platforms with optical [187,190,192,193] and electrical [188,189,191,192,194] responses and have shown prospective results for future investigation and application. For instance, in [187] and [193], SiNWs/GNP/AuNP and GO/AgNPs/Cu@Si substrates were utilized for R6G determination via SERS measurements. Additionally, it was found that GO modified AgNPs/Cu@Si substrates possessed higher SERS enhanced factor (2 × 10^12^) in comparison with bare AgNPs/Cu@Si (6,7 × 10^11^) [193]. This can be attributed to the well distributed hot spots and the GO films covering both AgNPs and spaces could make the probe molecule more effectively absorbed around the hot spots. While in the case of the absence of the GO film, the molecules will be distributed unevenly on the AgNPs/Cu@Si substrate, which will lead to the weak homogeneity of the SERS signal.

**Figure 9:**
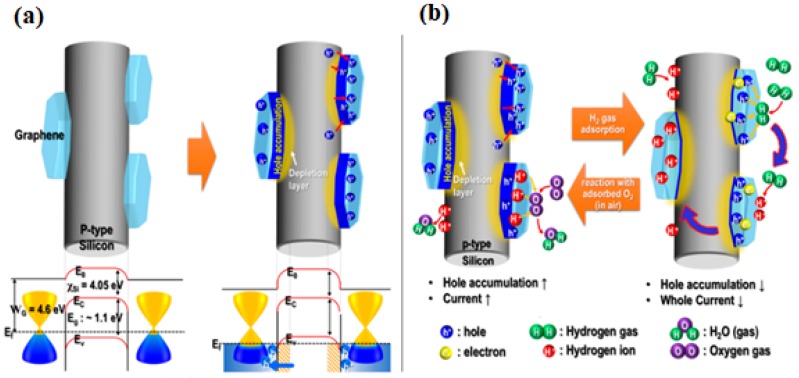
Schematic illustration showing (**a**) initial state of PSi/graphene substrate and formation of the depletion layer, (**b**) the adsorption-desorption process of H_2_ [194].

Eom et al. reported on PSi/graphene substrates suitable for room-temperature H_2_ gas detection via resistance measurements [194]. The main idea of this technique is similar to that of gas detection using nano-Si materials decorated with metal and/or MOx nanospecies. Generally, the sensing mechanism can be explained by the Schottky junction generation and formation of an electric depletion layer near the p-type Si and the hole accumulation layer near the graphene due to the difference in the Si and G work functions. Upon adsorption of the hydrogen gas molecules to the surface of the PSi/graphene, the accumulated holes near the graphene react with hydrogen molecules. As a consequence of this interaction, ionized hydrogen is formed, consequently leading to the reduction in the carrier density in the graphene layer. The conductivity of G-doped/p-Si becomes weaker due to the decreased graphene carrier concentration. Additionally, when the hydrogen gas was removed, the oxygen molecules in air react with the formed ionized hydrogen on the graphene and p-type Si, which increases the hole accumulation layer of graphene and decreases the ionized hydrogen in the p-type silicon, consequently, the conductivity of the PSi/graphene becomes higher (Figure 9).

Table 1 presents some of the main results on the application of nano-Si composites for (bio)sensor application. Table is divided into four sections, each of them corresponding to the nanostructures presented in Section 3, Section 4, Section 5 and Section 6.

## 7. Conclusions and Future Work

In this paper, we have provided an overview of the recent progress in (bio)sensing with nano-Si and nano-Si composites with polymers, MOx, metal nanoparticles, and carbon-based materials. It was found that novel nanocomposites are suitable for different detection techniques whereas pure nano-Si did not show acceptable results. For instance, pure nano-Si is hardly used for the SERS-based detection approach, while the nano-Si/MNps composites have recommended themselves as efficient SERS-active platforms with a high enhanced factor. Additionally, nano-Si, combined with the above-mentioned nanomaterials, possesses a number of different advantages such as the opportunity to obtain material with the necessary parameters and properties as well as using different surface (bio)functionalization protocols.

Significant attention has been paid to the estimation of gas sensing mechanisms. It should be noted that the nano-Si/MOx sensing mechanisms that have been provided in different publications have good correlation between each other and could be established as the fundamental knowledge in gas detection theory. Furthermore, novel sensing mechanisms have been proposed for more complicated nanostructures such as nano-Si/MOx/MNps. In this case, new effects are appearing and totally changing the type and rate of “sensor surface–gas” interaction.

Basic approaches and biosensing mechanisms that are now in use for nano-Si sensors have also been presented in detail. The advantages of this class of materials are that they can detect the target molecules in real-time with minimal sample damage and good repeatability. It can clearly be seen that researchers working in the area of improving the design and scheme of sensing equipment will gradually move to the size of microfluidic systems that possess a high precision of sample analysis. However, the fast response time, sensitivity, selectivity, long-term stability, and portable nano-Si based sensor devices remain important challenges for their future commercial applications.

To summarize the above-mentioned, there are many important challenges for the further prospective of nano-Si for fast and real-time diagnostic/detection. However, it can be clearly seen that all of the points of challenge could be solved through different approaches and techniques. For instance, filters can help to avoid the noise and background signal. A thick polymer layer coverage or combination of nano-Si with MOx, MNps, etc. could be used to achieve the nano-Si surface stability. The sensor’s signal homogeneity directly depends on the sensor’s surface homogeneity, which can be achieved by precise fabrication techniques such as electron beam lithography, photolithography, reactive ion lithography, etc. Microfluidic systems with incorporated nano-Si are the most prospective for the field of medicine and allows for the minimization of the necessary volume of detection solution. Other advantages of the microfluidic system are the small dimensions and the possibility of monitoring samples in real-time. The area of nano-Si sensor design is a multidisciplinary field, and many researchers are working on these challenges, furthermore, the rapid development of nanoscience and the appearance of novel tools will speed up the applied use of nano-Si.

## Figures and Tables

**Table 1:** Summarized data about nano-Si and nano-Si composites suitable for (bio)sensing applications.

**(Bio)sensors Based on PSi, SiNWs, SiNPs and Their Composites with Polymers**				
Type of transducer	Detection approach	Material for detection	LOD ^a^/Sensitivity ^b^ range/Sensitivity ^c^	Reference
PSi	Photoluminescence	Glucose, urea	^b^ 0–3.0 mM	[18]
		Cu^2+^, Pb^2+^, Cd^2+^	^c^ 10 nM	
	Colorimetric sensing	Autoimmune antibodies	^a^ 10 fg/mL	[73]
	Fluorescence	J774 macrophage cells	^a^ few and/or single cells	[75]
	Visual colorimetric sensing	Non-pathogenic *E. coli*	^a^ 1.5 ± 0.4 × 10^5^ CFU/mL	[100]
SiNWs	Resistance	H_2_O	^b^ 10–50 ppm	[117]
	Capacitance	Pressure	^a^ 0.1 Pa	[116]
	Luminescence	Streptavidin	^a^ 1.6 fM	[72]
	I–V curves	Near-infrared (NIR) light	^c^ 14.86–844.33 mA/W	[111]
SiNPLs	I–V curves	Relative humidity (RH)	^a^ 10%	[49]
	Refractive index	Isopropyl alcohol	^a^ 579.5 nm/RIU	[71]
	I–V curves	Ethanol, acetone gas	^a^ 0.25%	[108]
	I–V curves	Light	^c^ 1.3 mA/W	[109]
		UV light	^c^ 0.82 mA/W	
**(Bio)sensors based on nano-Si and MOx nanocomposites**				
PSi/WO_3_	Resistance	NO_2_	^a^ 100 ppb ^b^ 100 ppb–3 ppm	[129]
PSi/ZnO	Electrochemical impedance analysis	Ethanol solution	^b^ 0.05–0.6 M	[134]
PSi/TiO_2_	Fluorescence	Aflatoxins B1	^a^ 15.4 pg/mL	[26]
		Ochratoxin A	^a^ 1.48 pg/mL	
		Fumonisin B1	^a^ 0.21 pg/mL	
PSi/ZnO	Photocurrent	UV Light (325 nm)	^c^ 1.98 A/W	[123]
PSi/TiO_2_	I–V curve	UV illumination	^c^ 0.045 A/W	[132]
PSi/SnO_2_:Sn	Capacitance	Relative Humidity	^b^ 11–95%	[25]
SiNWs/TeO_2_/Pd	Resistance	C_6_H_6_, CO, C_7_H_8_, N_2_O	^b^ 10–50 ppm	[121]
SiNWs/ZnO	I–V curves	Glucose	^a^ 12 μM ^c^ 129 μA mM^−1^	[130]
SiNWs/WO_3_	Resistance	N_2_O	^b^ 0.25–5 ppm	[126]
SiNWs/ZnO	Resistance	N_2_O	^b^ 5–50 ppm	[131]
SiNPLs/Fe_2_O_3_/Ag	SERS	Malachite green (MG)	^a^ 10^−8^ M	[38]
SiNPLs/TiO_2_	I–V curves	CH_4_	^a^ 20 ppm	[136]
**(Bio)sensors based on nano-Si and metals nanoparticles**				
PSi/Ag	SERS	Rhodamine 6G	^a^ 10^−15^ M	[168]
		Crystal violet	^a^ 100 pM	[153]
		Porphyrin CuTMPyP4	^a^ 10^−11^ M	[151]
PSi/Au	Photoluminescence	Aflatoxin B1	^a^ 2.5 ± 0.5 pg/mL ^b^ 0.01–10 ng/ml	[13]
PSi/Ag	Amperometric response	Ascorbic acid	^a^ 0.83 μM ^c^ 1.279 mA mM^−1^ cm^−2^ ^b^ 20–600 μM	[170]
SiNWs/Au	Differential pulse voltammetry	DNA	^a^ 1.63 × 10^−12^ M	[160]
SiNWs/Ag	Resistance	NO_2_	^a^10 ppb	[27]
SiNWs/Au	I–V measurements	Glucose	^a^ 11 μM ^b^ 55.1 μM–16.53 mM	[148]
SiNWs/Au	Impedance measurements	Avidin	^a^ 10 × 10^−12^ M	[179]
SiNWs/Pd/Pt	Resistance	H_2_	^b^ 1–40,000 ppm	[181]
SiNPs/Au	SERS	Nerve gases VX	^a^ 13 fM	[12]
		Tabun	^a^ 630 fM	
SiNPs/Ag	SERS	Rhodamine 6G	^a^ 10^−11^ M	[177]
			^a^ 10^−13^ M ^b^ 10^−7^–10^−13^ M	[149]
SiNPs/Au	SERS	Rhodamine 6G	^b^ 10^−10^–10^−6^ M	[51]
		Cloxacillin	^b^ 15.6–500 pM	[183]
**(Bio)sensors based on nano-Si and carbon-based nanomaterials**				
PSi/GO substrate	Impedance	Aflatoxin B1	^b^ 1 fg/mL–1 pg/mL	[189]
PSi/GO AgNPs/PCu	SERS	Rhodamine 6 G	^a^ 10^−15^ M	[193]
PSi/Pd/GO	Resistance	H_2_	^a^ 200 ppm at 15 °C	[191]
PSi/Graphene	I–V curves	H_2_	^b^ 100–1000 ppm	[194]
SiNWs/Graphene	SERS	R6G	^a^ 10^−6^ M	[187]
SiNWs/Graphene	I–V curves characterization, PL measurements	DNA	^b^ 0.1–500 nM	[192]

Superscript letter **a**—indicates the limit detection (LOD), **b**—indicates sensor sensitivity range and **c**—indicates sensor sensitivity.
